# The antiSMASH database version 2: a comprehensive resource on secondary metabolite biosynthetic gene clusters

**DOI:** 10.1093/nar/gky1060

**Published:** 2018-11-05

**Authors:** Kai Blin, Victòria Pascal Andreu, Emmanuel L C de los Santos, Francesco Del Carratore, Sang Yup Lee, Marnix H Medema, Tilmann Weber

**Affiliations:** 1 Novo Nordisk Foundation Center for Biosustainability, Technical University of Denmark, Kemitorvet, Building 220, 2800 Kgs. Lyngby, Denmark; 2 Bioinformatics Group, Wageningen University, Wageningen, Netherlands; 3 Warwick Integrative Synthetic Biology Centre, University of Warwick, Coventry, UK; 4 Faculty of Science and Engineering, Manchester Institute of Biotechnology, University of Manchester, Manchester, UK; 5 Department of Chemical and Biomolecular Engineering (BK21 Plus Program), Korea Advanced Institute of Science and Technology (KAIST), Daejeon, Republic of Korea

## Abstract

Natural products originating from microorganisms are frequently used in antimicrobial and anticancer drugs, pesticides, herbicides or fungicides. In the last years, the increasing availability of microbial genome data has made it possible to access the wealth of biosynthetic clusters responsible for the production of these compounds by genome mining. antiSMASH is one of the most popular tools in this field. The antiSMASH database provides pre-computed antiSMASH results for many publicly available microbial genomes and allows for advanced cross-genome searches. The current version 2 of the antiSMASH database contains annotations for 6200 full bacterial genomes and 18,576 bacterial draft genomes and is available at https://antismash-db.secondarymetabolites.org/.

## INTRODUCTION

A majority of antibacterial and antifungal drugs, as well as drugs for many other indications, are derived from microbial natural products (1). Traditionally, bioactive natural compounds were identified via classical isolation and analysis approaches. The increasing availability of genomic data in the last two decades allows us to complement these approaches with genome mining to identify and characterize biosynthetic pathways for natural products in genome and metagenome data (2). Specialized software to support researchers in their search for natural products has been available for some years (for a comprehensive overview/list of such tools, please see (3–5)). Since its initial release in 2011, antiSMASH (6–9) has established itself as a standard tool for secondary metabolite genome mining and is currently the most widely used software pipeline for this task.

antiSMASH uses a rule-based cluster detection approach to identify 45 different types of secondary metabolite biosynthetic pathways via their core biosynthetic enzymes. For nonribosomal peptide synthases, type I polyketides, terpenes, lanthipeptides, thiopeptides, sactipeptides and lassopeptides, antiSMASH can also provide more detailed predictions of the compounds produced by the respective biosynthetic gene clusters (BGCs). Identified clusters are compared to a database of clusters previously predicted by antiSMASH using the built-in ClusterBlast algorithm. A similar algorithm, KnownClusterBlast is used to compare the identified cluster against the manually curated set of known BGCs from the MIBiG (10) database. Secondary metabolite clusters of orthologous group (smCoG) classification is used to assign functions to gene products in the predicted BGCs.

As antiSMASH is a genome mining pipeline designed to analyze individual genomes, we developed the antiSMASH database (11) to provide interconnections and cross-genome search functionality based on antiSMASH results for many publicly available microbial genomes. Moreover, it provides users with instant access to full antiSMASH results of publicly available genome sequences. Here we present version 2 of the antiSMASH database. The database content of version 1, which was generated with version 3 of antiSMASH, was updated with annotation of the current antiSMASH 4.2.1 release. This implies that the antiSMASH database now includes updated detection rules, updated ClusterBlast database links, TTA codon prediction, NRPS-A domain predictions by the up-to-date SANDPUMA software (12), classification of terpenes and improved links to MIBiG (10) (for details, please see (9)). Furthermore, new sequences that became available after version 1 release were included. Version 2 of the antiSMASH database now contains genome mining results for 6,200 full bacterial genomes and 18 576 draft genomes from the NCBI RefSeq database (13). The increased dataset is accompanied by improvements in the search functionality, data export options and the user interface of the antiSMASH database.

## MATERIALS AND METHODS

### Selection of included genomes

Microbial genome resources are growing rapidly and, despite taxonomically novel genomes being released frequently, there is a lot of sequence redundancy in the NCBI genome databases, i.e. thousands of sequences of mostly pathogenic bacteria such as *Pseudomonas aeruginosa* or *Escherichia coli*. Therefore, with the objective of creating a representative set of genomes that are non-redundant, we designed an approach to effectively update the antiSMASH database, maintaining its high quality and adequately representing natural diversity without significantly decreasing the overall pipeline performance in terms of speed.

Genomes categorized as ‘draft genomes’ are fragmented in multiple contigs. As many secondary metabolite biosynthetic gene cluster contain repetitive sequences, this implies that many BGCs end up being split on multiple contigs without any linkage information, leading to low-quality BGC data. Consequently, in order to minimize this issue we prioritized the inclusion of NCBI RefSeq genomes that were annotated with the assembly level ‘complete genome’ or ‘chromosome’ present in the database on April 2018 (10 863 genomes in total). We then estimated the distance between selected assemblies using fastANI (Average Nucleotide Identity) (https://github.com/ParBLiSS/FastANI). FastANI uses a hash-based algorithm to estimate the average nucleotide identity between pairs of genomic assemblies. A network was generated with each genome as a node, and weighted edges between nodes corresponding to the fastANI estimate between genomes. We used a fastANI similarity score of 99.6 as a cutoff for having an edge between nodes. Nodes were then assigned to communities using the multilevel community structure algorithm (https://arxiv.org/abs/0803.0476) in the igraph Python package (Csardi G, Nepusz T: The igraph software package for complex network research, InterJournal, Complex Systems 1695. 2006. http://igraph.org). Finally, a representative genome from each community was chosen by prioritizing assemblies with the highest contig N50 and lowest contig L50. This resulted in a total of 6,200 complete genomes for the antiSMASH database.

In order to supplement the set of complete and chromosomal assemblies, we added a set of draft genomes to the antiSMASH database. To select draft genomes for addition to the database, we started with a previously published set of precomputed fastANI similarity scores of ninety thousand prokaryotic genomes (https://doi.org/10.1101/225342). We pre-filtered this set to remove poor quality genomes (N50 < 20 kb and assembly anomalies). We then performed the same procedure as with the complete and chromosomal assemblies to group the draft genomes into communities. A representative genome from each community was chosen by prioritizing assemblies based on assembly level (scaffold > contig), and then selecting assemblies with the highest contig N50 and lowest contig L50. In order to maintain consistency with the complete and chromosomal set, only draft genomes that had corresponding RefSeq assemblies were included in the database. The following resulted in an additional 18,576 draft genome entries that were added to the database.

### antiSMASH annotations and data import

Based on the selection criteria mentioned above, the assemblies were downloaded from the NCBI servers in GenBank format using the ncbi-genome-download tool (https://github.com/kblin/ncbi-genome-download/). GNU parallel (14) was used to run multiple docker containers of antiSMASH 4.2.1 simultaneously. Different analysis parameters were used for the full and partial genome set. For full genomes, ClusterBlast, KnownClusterBlast, SubClusterBlast, ActiveSiteFinder, TTA codon detection in automatic mode, secondary metabolite clusters of orthologous groups prediction, and cluster-specific detailed annotations were run (command line flags: --clusterblast --knownclusterblast --subclusterblast --asf --tta-auto --smcogs-notree). For draft genomes, antiSMASH was run in fast mode, skipping the detailed annotations. Additionally, KnownClusterBlast, TTA codon detection in automatic mode, and secondary metabolite clusters of orthologous groups prediction were run (command line flags: --minimal --knownclusterblast --tta-auto --smcogs-notree).

The SQL schema of the (https://github.com/antismash/db-schema/) antiSMASH database was updated to accommodate the annotation changes and additional features/predictions that were introduced by antiSMASH version 4. The antiSMASH results in GenBank format were loaded into the SQL schema using the import script available at https://github.com/antismash/db-import/.

## RESULTS AND DISCUSSION

With an update to the PGAP annotation pipeline used by the NCBI, the annotation issues causing us to use records from GenBank instead of RefSeq for version 1 of the antiSMASH database have largely been resolved. Hence, with version 2 of the database, we have switched to using RefSeq genomes to obtain more unified gene annotations.

The antiSMASH database 2 contains BGCs identified in 6,200 full genomes (an increase of 58%) and adds 18 576 draft genomes. Annotations in the database are generated by antiSMASH version 4.2.1, the most recent release of antiSMASH (9). New in the antiSMASH 4.2.1 release are detection rules for *N*-acyl amino acids, polybrominated diphenyl ethers, and PPY-like pyrones. Detailed cluster product predictions have been added for lasso peptides, thiopeptides, sactipeptides (based on RODEO (15)), non-ribosomal peptide synthases (based on SANDPUMA (12)) and terpenes. The ClusterBlast and KnownClusterBlast databases have been updated.

The search builder has been extended to cover these new features. A new search field in the taxonomy browser makes it easier to navigate to species of interest in the much larger dataset.

The gene cluster data obtained in the queries can be downloaded. Depending on the type of search, different file formats are available. For gene cluster searches, the result table can be downloaded in tabular (CSV) format, alternatively it is possible to retrieve the DNA sequence of all matching clusters in FASTA format. Gene and protein domain searches offer a download the protein and nucleotide sequences of all matching genes or protein domains, respectively, or a tabular representation of the results. New options are provided to download specific chunks of the result data (for example only the first 1000 sequences) and to select between standard FASTA headers including the IDs and descriptive headers also including the query the hits were obtained with.

The selection of genomes available from NCBI still skews the perspective on the available diversity of biosynthetic gene clusters. While the antiSMASH database contains sequences from 33 different phyla, sequences from e.g. proteobacteria are vastly overrepresented due to their significance as pathogens. The database now contains 32 548 biosynthetic gene clusters from the full genome dataset, an increase of 46% from version 1 (Table 1). Statistics from the 18 576 draft genomes certainly overpredict the number of identified clusters due to clusters being split over several contigs and counted multiple times, the fast-mode results still provide a good first estimate of the available biosynthetic diversity of the draft genomes. Of the 119 558 BGCs predicted on the draft genomes, over a third (41 482) are in contact with at least one contig edge and thus likely incomplete. In comparison, only ∼1% of the clusters from the full genome dataset (390 in 32 548) are located on a contig edge. As the abundant fragmentation of clusters in draft genomes is skewing the numbers, the following statistics only count the results from the full genomes. See Table 2 for detailed cluster counts by BGC type and a comparison with the cluster counts from version 1.

**Table 1.** Overview on BGC numbers in version 1 and version 2 of the antiSMASH database

Overall database statistics	Version 1 counts	Version 2 counts	% change
**Full (high quality) genomes**	3907	6200	58
**Number of BGCs in full genomes**	22 292	32 548	46
**Draft genomes**	0	18 576	New
**Number of BGCs in draft genomes**	0	119 558	New
**BGCs in total**	22 292	152 106	682

**Table 2.** Changes in cluster counts of the different BGC types between version 1 and version 2 of the antiSMASH database (excluding data from draft genomes)

Gene cluster types (high quality genomes)	Version 1 counts	Version 2 counts	% change
**NRPS**			
Nonribosomal peptide	5878	7893	34
**Terpenes**			
Terpene	3362	5018	49
**Polyketides**			
Type I polyketide	2608	3302	27
Type III polyketide	742	1141	54
hglE-type polyketide	590	768	30
Trans-AT polyketide	512	623	22
Type II polyketide	173	307	77
PPY-like pyrone	0	13	New
**RiPPs**			
Bacteriocin/RiPP	3323	5198	56
Lanthipeptide	857	1121	31
Thiopeptide	122	1097	799
Lasso peptide	351	562	60
Sactipeptide	59	318	439
Microviridin	18	70	289
Head-to-tail cyclised (subtilosin-like)	22	52	136
Proteusin	13	39	200
Microcin	5	3	–40
Bottromycin-like	1	2	100
**Other**			
Other	1887	2322	23
Siderophore	1399	1745	25
Homoserine lactone	1084	1608	48
Aryl polyene	988	1595	61
Ectoine	424	794	87
Butyrolactone	189	392	107
Phosphonate	248	342	38
Resorcinol	184	261	42
Ladderane	113	217	92
Phenazine	152	210	38
Melanin	45	113	151
*N*-acyl amino acid cluster	0	110	New
Indole	48	104	117
Cyanobactin	30	77	157
Polyunsaturated fatty acid	45	61	36
Oligosaccharide	40	54	35
Aminoglycoside/aminocyclitol	26	51	96
Nucleoside	23	49	113
Linaridin	17	35	106
beta-lactam	13	30	131
Aminocoumarin	3	10	233
Pheganomycin-like ligase	5	7	40
Phosphoglycolipid	1	4	300
Furan	2	3	50
Glycocin	14	3	–79
Polybrominated diphenyl ether	0	1	New

**Figure 1.**
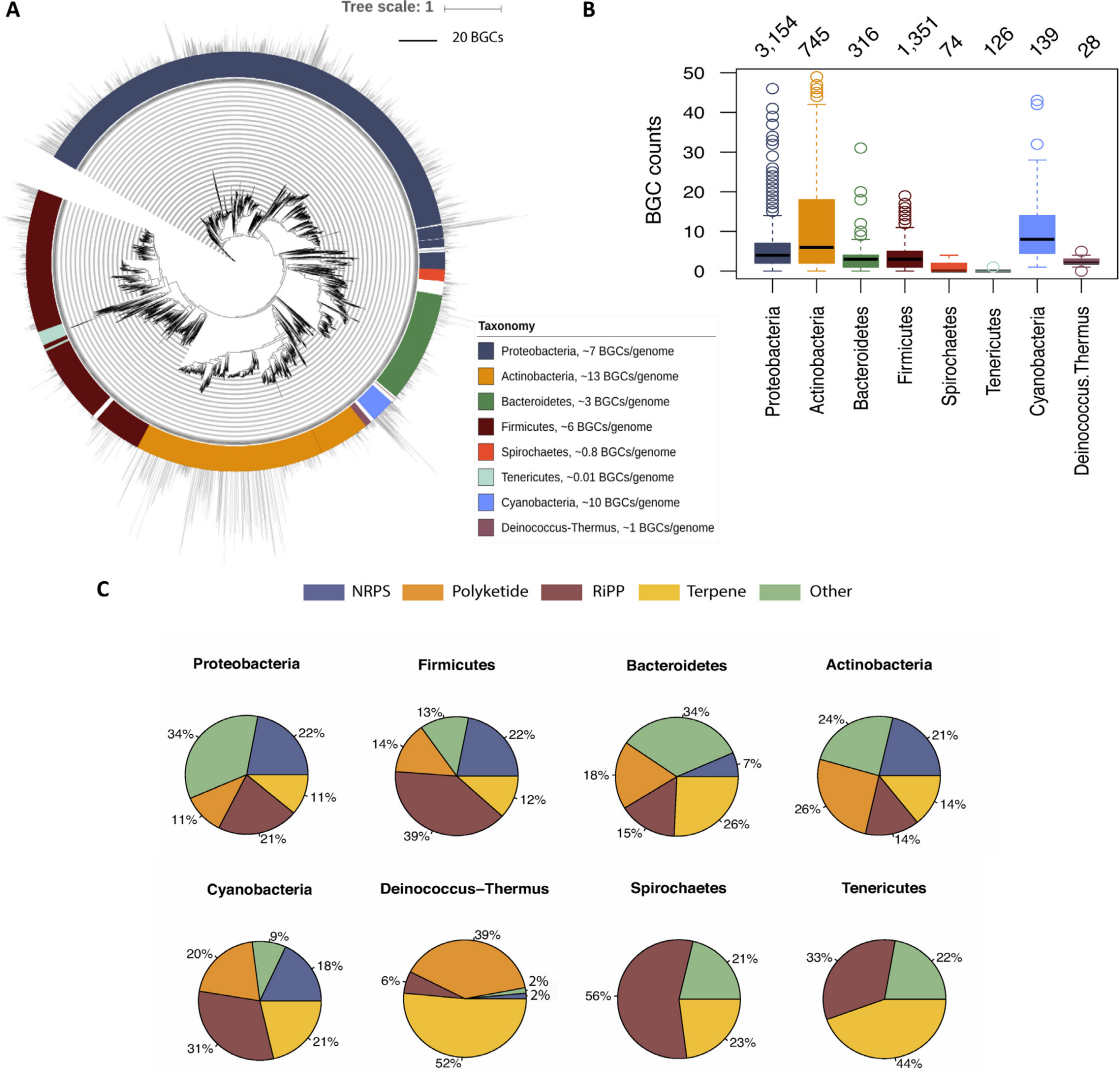
Statistic summary of the antiSMASH database version 2. (**A**) A phylogenetic tree constructed from the revised version of tree of life based on 120 conserved protein markers (16). The original tree was pruned by genome assembly id using ETE Toolkit (17), to only keep leaves that belong to genomes of the antiSMASH database version 2. The visualization and customization of the tree was performed with iTOL (18). As a result, 12 219 leaves from the total of 24,776 bacterial genomes are shown in this phylogeny. The colored ring represents the eight most abundant phyla; 97.6% of the genomes, and the bar plots in the outer ring the number of BGCs per genome. (**B**) Boxplots of the BGCs counts per phylum, with the values on top showing the total number of complete genomes per phylum. (**C**) Pie charts of the five major BGC classes per phylum showing the diversity of natural products produced by each group of bacteria.

In order to get an accurate taxonomic overview, the identified BGCs were mapped to a phylogenetic tree displaying approximately half of the genomes (12 219 complete and draft) that are included in the database (Figure 1A). The topology of the tree shows the microbial diversity chosen, ranging from well characterized phyla to unclassified bacteria found in diverse ecosystems. Proteobacteria, Actinobacteria, Bacteroidetes, Firmicutes, Spirochaetes, Tenericutes, Cyanobacteria and *Deinoccocus-Thermus*, the eight most abundant bacterial divisions in our database, accounting for 97.6% of genomes and all vary in the number of harbored BGCs (Figure 1B). High BGC numbers are characteristic features for some groups of bacteria such as *Actinobacteria* (containing 13 clusters on average (full genomes) while others rarely possess one, like *Tenericute*. These bacteria exhibit different distributions in terms of encoded secondary metabolite types as defined by antiSMASH (Figure 1C). For these statistics, the 45 BGC classes in antiSMASH have been condensed into five major groups: Non-Ribosomal Peptide Synthetase (NRPS), Polyketide, Ribosomally synthesized and post-translationally modified peptides (RiPP), terpenes and Others, clusters that do not belong to any of the aforementioned types. Terpenes, bacteriocins (a type of RiPP) and NRPS are the most common BGC types, all with higher number of representatives in the phylum *Proteobacteria*.

## CONCLUSIONS

Genome mining is a valuable method to assess the biosynthetic potential of microorganisms. Since 2011, antiSMASH has assisted researchers with their secondary metabolite genome mining projects. The public web service has processed ∼400 000 jobs, and the standalone tool has been downloaded over 10 000 times. The antiSMASH database both allows instant access to antiSMASH results for many publicly available genomes instead of waiting several hours for a de-novo antiSMASH run and allows advanced cross-genome searches for BGCs with specific features of interest.

In comparison to version 1, the updated version 2 of the antiSMASH database provides antiSMASH 4.2.1 annotations for 6200 full genomes, which is an increase by 58%, and newly introduces data for 18 576 draft genomes. The graphical query builder allows researchers to interactively formulate searches to answer cross-genome research questions, while the results are presented in the familiar antiSMASH output format.

## DATA AVAILABILITY

The antiSMASH database is available at https://antismash-db.secondarymetabolites.org/. There are no access restrictions for academic or commercial use of the web server. The source code components and SQL schema for the antiSMASH database are available on GitHub (https://github.com/antismash) under an OSI-approved Open Source license.
